# Supplementary material for: The level of oncogenic Ras determines the malignant transformation of Lkb1 mutant tissue in vivo
Source: Commun Biol. 2021 Jan 29;4:142. doi: 10.1038/s42003-021-01663-8 (PMC7846793; doi:10.1038/s42003-021-01663-8)

Supplementary Information

for

The level of oncogenic Ras determines the malignant transformation of Lkb1 mutant tissue in vivo

Briana Rackley, Chang-Soo Seong Evan Kiely, Rebecca E. Parker, Manali Rupji, Bhakti Dwivedi[4] John M. Heddleston, William Giang, Neil Anthony, Teng-Leong Chew & Melissa Gilbert-Ross

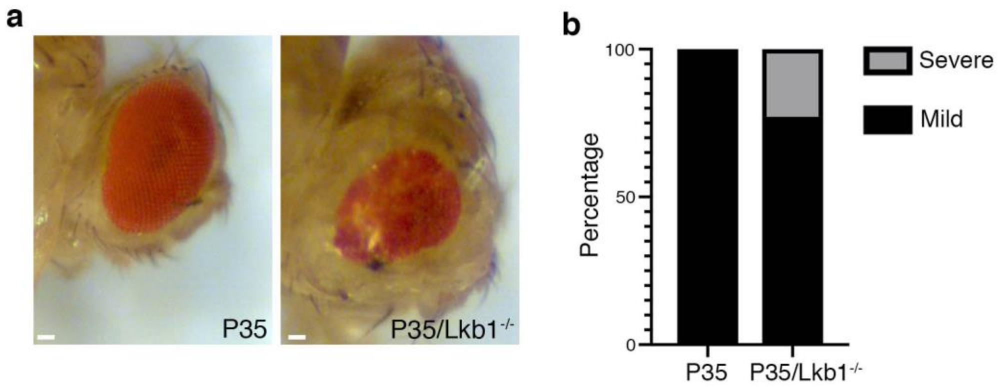

Supplementary Figure 1. Blocking cell death with *P35* in *Lkb1* mutant clones does not phenocopy *Ras*^{Lo}*/Lkb1*$^{-/-}$. (a) Brightfield images of mosaic adult eyes expressing *P35* (left) or *P35/Lkb1*$^{-/-}$ (right). Scale bar, 20µm. (b) Percentage of *P35* or *P35/Lkb1*$^{-/-}$ mosaic eyes with either a mild or severe phenotype (severe phenotype is pictured in (a) for *P35/Lkb1*$^{-/-}$.

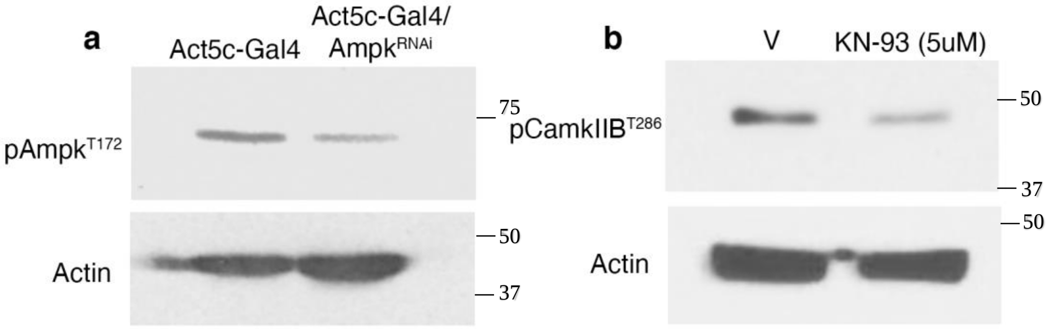

Supplementary Figure 2. Validation of genetic and pharmacologic reagents. (a) Western analysis of activated Drosophila Ampk from whole larvae of the indicated genotypes. (b) Western analysis of activated Drosophila pCamkIIB from eye/imaginal disc tumors from Ras^{Hi}/Lkb1$^{-/-}$ larvae treated with vehicle or KN-93- (5uM).

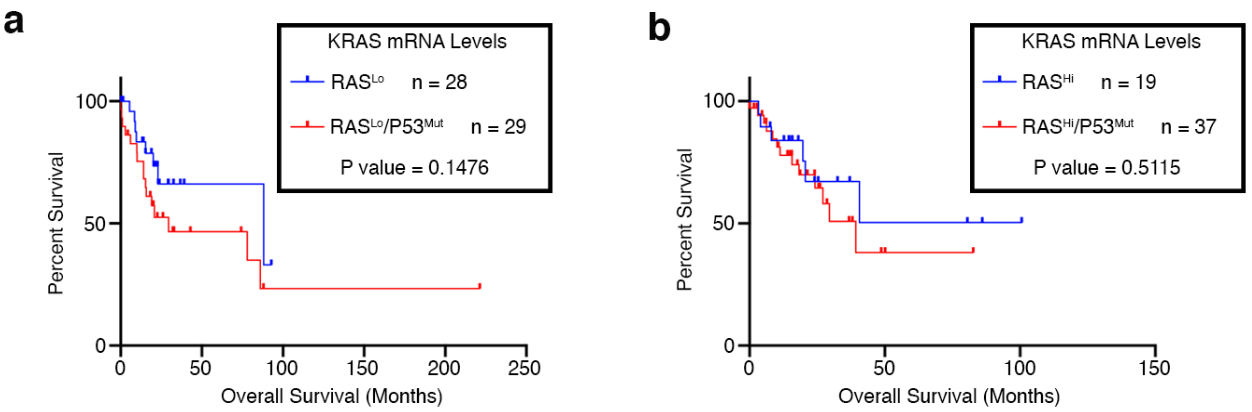

Supplementary Figure 3. High level *KRAS* does not result in survival differences in *TP53* mutant lung cancer patients. (a-b) Survival analysis using the TCGA Pan Lung Cancer study. Patients were stratified as RAS^{Lo} or RAS^{Hi} using *KRAS* mRNA expression and further stratified based on *TP53* deletion and loss-of-function mutation status. Data were graphed using a Kaplan-Meier survival plot.

Supplementary Figure 4

Originals Figure 1a

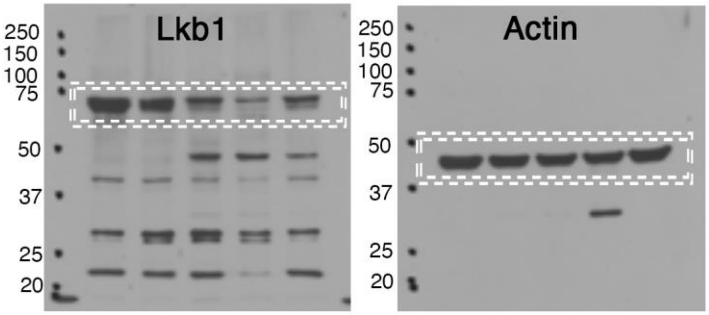

Originals Figure 1b

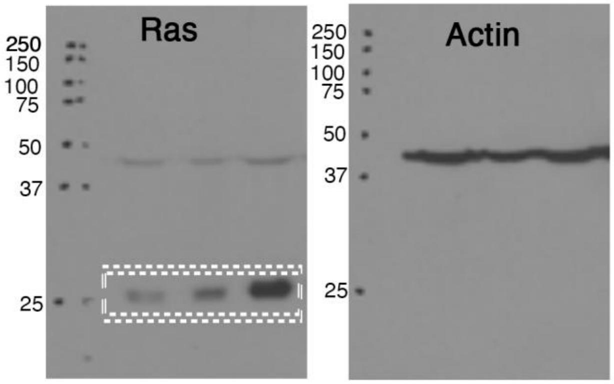

Supplementary Figure 5

Original data Figure 5a

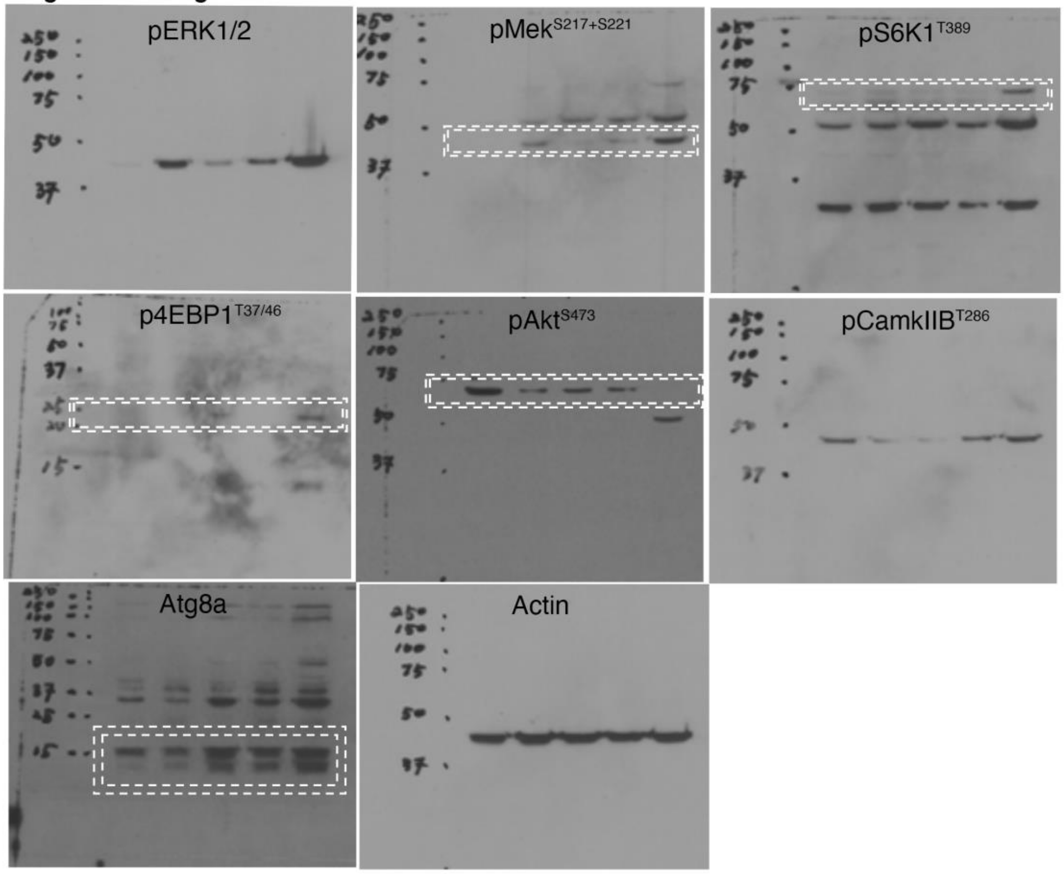

Original data Figure 5b

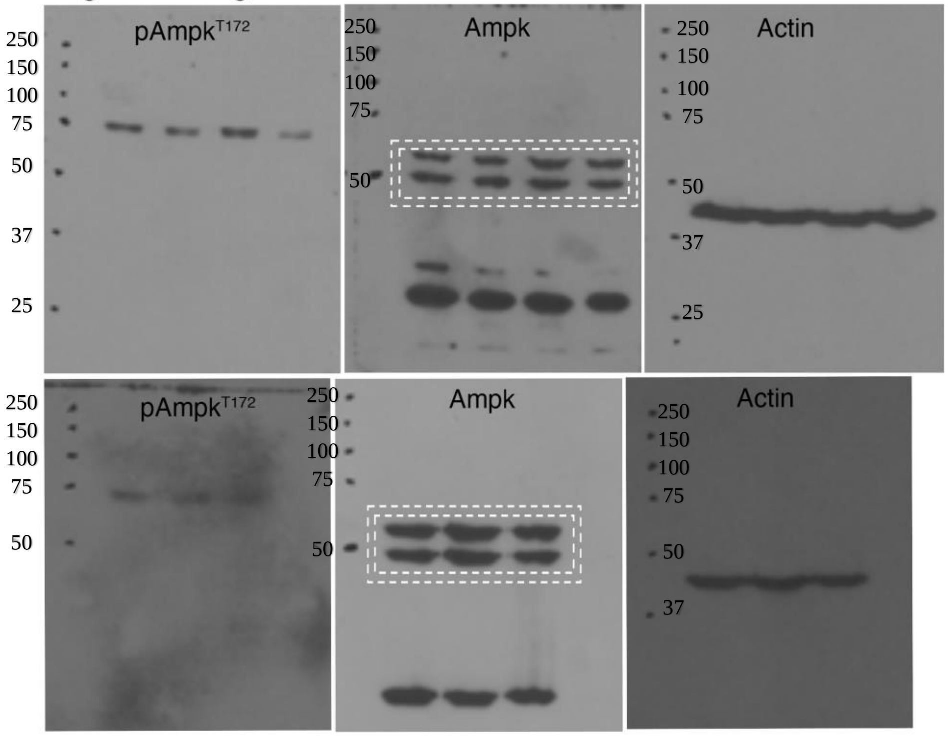

Supplementary Figure 6

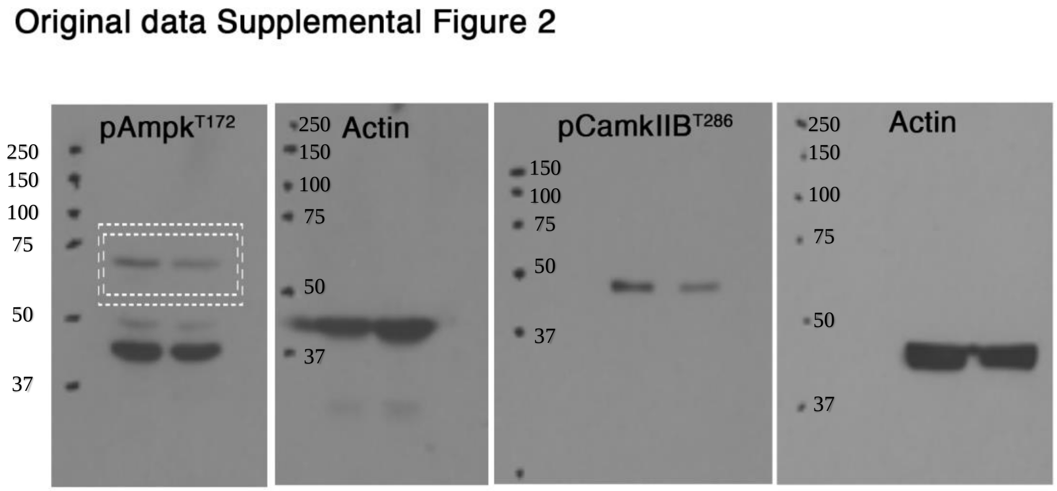

Supplement: Supplementary file 2 — Supplemental Information [file 42003_2021_1663_MOESM2_ESM.pdf]
