# Supplementary material for: The level of oncogenic Ras determines the malignant transformation of Lkb1 mutant tissue in vivo
Source: Commun Biol. 2021 Jan 29;4:142. doi: 10.1038/s42003-021-01663-8 (PMC7846793; doi:10.1038/s42003-021-01663-8)
Supplement: Supplementary file 3 — Description of Supplementary Files [file 42003_2021_1663_MOESM3_ESM.pdf]

Description of Additional Supplementary Files

File name: Supplementary Movie 1

Description: Long-term imaging of live tumor cell invasion into collagen in Ras^{V12}/*$Lkb1^{-/-}$* giant larvae.

File name: Supplementary Data 1

Description: Source data for quantification of DCP1 staining.

File name: Supplementary Data 2

Description: Source data for DNA content and cell cycle analysis.

File name: Supplementary Data 3

Description: Source data for allograft survival analysis.

File name: Supplementary Data 4

Description: Source data for viability assays after drug treatment.
