# Supplementary material for: The level of oncogenic Ras determines the malignant transformation of Lkb1 mutant tissue in vivo
Source: Commun Biol. 2021 Jan 29;4:142. doi: 10.1038/s42003-021-01663-8 (PMC7846793; doi:10.1038/s42003-021-01663-8)
Supplement: Supplementary file 9 — Reporting Summary [file 42003_2021_1663_MOESM9_ESM.pdf]

nature research

Corresponding author(s): Melissa Gilbert-Ross

Last updated by author(s): Nov 20, 2020

Reporting Summary

Nature Research wishes to improve the reproducibility of the work that we publish. This form provides structure for consistency and transparency in reporting. For further information on Nature Research policies, see Authors & Referees and the Editorial Policy Checklist.

Statistics

For all statistical analyses, confirm that the following items are present in the figure legend, table legend, main text, or Methods section.

n/a	Confirmed	
☐	☒	The exact sample size (*n*) for each experimental group/condition, given as a discrete number and unit of measurement
☐	☒	A statement on whether measurements were taken from distinct samples or whether the same sample was measured repeatedly
☐	☒	The statistical test(s) used AND whether they are one- or two-sided<br>*Only common tests should be described solely by name; describe more complex techniques in the Methods section.*
☐	☒	A description of all covariates tested
☐	☒	A description of any assumptions or corrections, such as tests of normality and adjustment for multiple comparisons
☐	☒	A full description of the statistical parameters including central tendency (e.g. means) or other basic estimates (e.g. regression coefficient) AND variation (e.g. standard deviation) or associated estimates of uncertainty (e.g. confidence intervals)
☐	☒	For null hypothesis testing, the test statistic (e.g. *F*, *t*, *r*) with confidence intervals, effect sizes, degrees of freedom and *P* value noted<br>*Give P values as exact values whenever suitable.*
☒	☐	For Bayesian analysis, information on the choice of priors and Markov chain Monte Carlo settings
☒	☐	For hierarchical and complex designs, identification of the appropriate level for tests and full reporting of outcomes
☐	☒	Estimates of effect sizes (e.g. Cohen's *d*, Pearson's *r*), indicating how they were calculated

Our web collection on statistics for biologists contains articles on many of the points above.

Software and code

Policy information about availability of computer code

Data collection	No software was used for collection of data.
Data analysis	FIJI (ImageJ) and IMARIS software were used for analysis of data obtained using microscopy. For faster image rendering, custom code was created to automatically create maximum intensity projections of SIMView data. FlowJo software was used for analysis of cell cycle data. GraphPad PRISM and R software were used for statistical analysis. HiPathia software was used for Circuit analysis.

For manuscripts utilizing custom algorithms or software that are central to the research but not yet described in published literature, software must be made available to editors/reviewers. We strongly encourage code deposition in a community repository (e.g. GitHub). See the Nature Research guidelines for submitting code & software for further information.

Data

Policy information about availability of data

All manuscripts must include a data availability statement. This statement should provide the following information, where applicable:

- Accession codes, unique identifiers, or web links for publicly available datasets
- A list of figures that have associated raw data
- A description of any restrictions on data availability

The molecular and clinical data used to support the conclusions of Figure 6 are available from the GDC data portal (http://portal.gdc.cancer.gov/). Source data for Figure 1e, Figure 2b,c, Figure 3f, and Figure 5g,h are available with the paper. All other data that support the findings of this paper are available from the corresponding author upon request.

Field-specific reporting

Please select the one below that is the best fit for your research. If you are not sure, read the appropriate sections before making your selection.

☒ Life sciences ☐ Behavioural & social sciences ☐ Ecological, evolutionary & environmental sciences

For a reference copy of the document with all sections, see nature.com/documents/nr-reporting-summary-flat.pdf

Life sciences study design

All studies must disclose on these points even when the disclosure is negative.

Sample size	No sample size calculations were performed prior to the study. All sample sizes are indicated in the figure legends.
Data exclusions	For TCGA analysis, one patient was excluded as an outlier.
Replication	For microscopy, multiple independent biological samples were imaged for reproducibility. For FACS and Western analysis , 3 independent biological replicates were performed. For SIMView data, 2 independent larvae were imaged (experiment is terminal). Drug experiments were replicated twice and genetic rescue was performed using 3 biological replicates.
Randomization	Samples and organisms were randomly selected for all treatments, experiments, and imaging.
Blinding	Blinding was not used for our study, as no data were obtained where blinding would be necessary or beneficial.

Reporting for specific materials, systems and methods

We require information from authors about some types of materials, experimental systems and methods used in many studies. Here, indicate whether each material, system or method listed is relevant to your study. If you are not sure if a list item applies to your research, read the appropriate section before selecting a response.

Materials & experimental systems

n/a	Involved in the study	
☐	☒	Antibodies
☒	☐	Eukaryotic cell lines
☒	☐	Palaeontology
☐	☒	Animals and other organisms
☒	☐	Human research participants
☒	☐	Clinical data

Methods

n/a	Involved in the study	
☒	☐	ChIP-seq
☒	☐	Flow cytometry
☒	☐	MRI-based neuroimaging

Antibodies

Antibodies used	rabbit anti-cleaved Drosophila DCP1 (Asp216) (Cell Signaling, 1:100), mouse anti-MMP1 (3A6B4/5H7B11/3B8D12 antibodies were mixed in equal amounts) (DSHB, 0.2µg/ml), rabbit anti-pAMPK (T172) (Cell Signaling 1:100), Alexa Fluor 488 (Life Technologies), Alexa Fluor 555 (Life Technologies), Alexa Fluor 647 (Life Technologies), affinity purified guinea pig anti-Drosophila Lkb1 (Protein Tech, 1:1000), rabbit anti-Ras (Cell Signaling 3965, 1:1000), rabbit anti-phospho AMPK (Thr 172) (40HP) (Cell Signaling, 1:1000), mouse anti-Drosophila ampk1/2 (BioRad, 1:1000), rabbit anti-diphosphorylated ERK (Sigma, 1:1000), Rabbit anti-phospho MEK1 (Ser 217+221) (Invitrogen, 1:500), rabbit anti-Drosophila phospho p70 S6 Kinase (Thr 398) (Cell Signaling (1:1000), rabbit anti-phospho 4E-BP1 (Thr 37/46) (Cell Signaling, 1:1000), rabbit anti-phospho AKT (Ser 473) (Cell Signaling, 1:1000), mouse anti-phospho CaMKII (Thr 286) (22B1 Santa Cruz Biotechnology, 1:200), rabbit anti-ATG8a (Creative Diagnostics, 0.2g/ml), mouse anti-actin (JLA20) (Developmental studies Hybridoma Bank, 1:1000), and Hoechst 33342 (Cell Signaling, 500 g/ml).
Validation	Each commercially acquired primary antibody was validated based on specific statements from the manufacturers website. For the affinity purified guinea pig anti-Drosophila Lkb1 antibody generated using Protein Tech, the antibody was validated using a panel of specific Drosophila lines as negative and positive controls. This data is provided in the manuscript, along with how the antibody was generated.

Animals and other organisms

Policy information about studies involving animals; ARRIVE guidelines recommended for reporting animal research

Laboratory animals	This study used both male and female Drosophila Melanogaster at all stages of development, from embryo to adult. The following strains were used: FRT82B, w1118;df(3R)Exel6169,P{XP-U}Exel6169/TM6B,Tb, UAS-RasV12; FRT82B, UAS-RasV12,

	FRT82B, UAS-AmpkTrip20(RNAi), w1118, Viking-GFP, Lkb14A4-2, Lkb14B1-11, Lkb1X5, y,w, eyFLP1; Act >y+> Gal4, UAS-GFP (or RFP); FRT82B, Tub-Gal80.
Wild animals	This study did not involve wild animals.
Field-collected samples	This study did not involve samples collected from the field.
Ethics oversight	No ethical approval or guidance was required for this study, as Drosophila are not a USDA regulated organism.

Note that full information on the approval of the study protocol must also be provided in the manuscript.
